# Adaptive Responses to Oxidative Stress in the Filamentous Fungal *Shiraia bambusicola*

**DOI:** 10.3390/molecules21091118

**Published:** 2016-08-24

**Authors:** Huaxiang Deng, Jiajun Chen, Ruijie Gao, Xiangru Liao, Yujie Cai

**Affiliations:** The Key Laboratory of Industrial Biotechnology, Ministry of Education, School of Biotechnology, Jiangnan University, 1800 Lihu Road, Wuxi 214122, Jiangsu, China; denghxiang@163.com (H.D.); 7140201017@vip.jiangnan.edu.cn (J.C.); gaoruijie1989@163.com (R.G.)

**Keywords:** oxidative stress, adaptive responses, hypocrellin biosynthesis, hydrogen peroxide, *Shiraia bambusicola*, filamentous fungi

## Abstract

*Shiraia bambusicola* can retain excellent physiological activity when challenged with maximal photo-activated hypocrellin, which causes cellular oxidative stress. The protective mechanism of this fungus against oxidative stress has not yet been reported. We evaluated the biomass and hypocrellin biosynthesis of *Shiraia* sp. SUPER-H168 when treated with high concentrations of H_2_O_2_. Hypocrellin production was improved by nearly 27% and 25% after 72 h incubation with 10 mM and 20 mM H_2_O_2_, respectively, while the inhibition ratios of exogenous 20 mM H_2_O_2_ on wild *S. bambusicola* and a hypocrellin-deficient strain were 20% and 33%, respectively. Under exogenous oxidative stress, the specific activities of catalase, glutathione reductase, and superoxide dismutase were significantly increased. These changes may allow *Shiraia* to maintain normal life activities under oxidative stress. Moreover, sufficient glutathione peroxidase was produced in the SUPER-H168 and hypocrellin-deficient strains, to further ensure that *S. bambusicola* has excellent protective abilities against oxidative stress. This study creates the possibility that the addition of high H_2_O_2_ concentrations can stimulate fungal secondary metabolism, and will lead to a comprehensive and coherent understanding of mechanisms against oxidative stresses from high hydrogen peroxide concentrations in the filamentous fungal *Shiraia* sp. SUPER-H168.

## 1. Introduction

*Shiraia bambusicola* is known as a pathogenic fungus of bamboo species, including Brachystachyum densiflorum [1] in China and Bambusa sp. in Japan [2]. *S. bambusicola* also has the ability to produce hypocrellin, which is a kind of perylenequinone [3]. In addition, hypocrellin can generate reactive oxygen species (ROS) when it interacts with molecular oxygen during illumination [4]. These ROS include singlet oxygen, superoxide radicals, and hydroxyl radicals. Based on these characteristics, hypocrellin has been widely used as a photosensitizer for medical purposes, such as photodynamic tumor therapy and antivirus treatment [5,6,7]. In addition, hypocrellin has been applied in the treatment of skin diseases for many years in China.

Most filamentous fungi can inevitably generate H_2_O_2_ because of an incomplete reduction of oxygen during respiration [8]. The less deleterious H_2_O_2_ can be potentially transformed into higher toxic hydroxyl radicals by the Fenton reaction [9]. These hydroxyl radicals may aggravate cellular damage. The redundant ROS in aerobic cells will cause cellular oxidative stresses and be irreversibly harmful to macromolecules, including lipids, proteins, and DNA [10]. However, cells can still retain redox balances and normal morphology via their cellular antioxidant systems [10]. Cai et al. reported that the fungal *Shiraia* sp. SUPER-H168 can retain remarkable morphology and high biomasses even in plentiful hypocrellin production [11], which can produce light-activated oxidative stresses. It is suggested that this *S. bambusicola* has an excellent antioxidant system against oxidative stress from abundant ROS. However, the defense mechanism of *S. bambusicola* against oxidative stress has not yet been reported.

**Figure 1:**
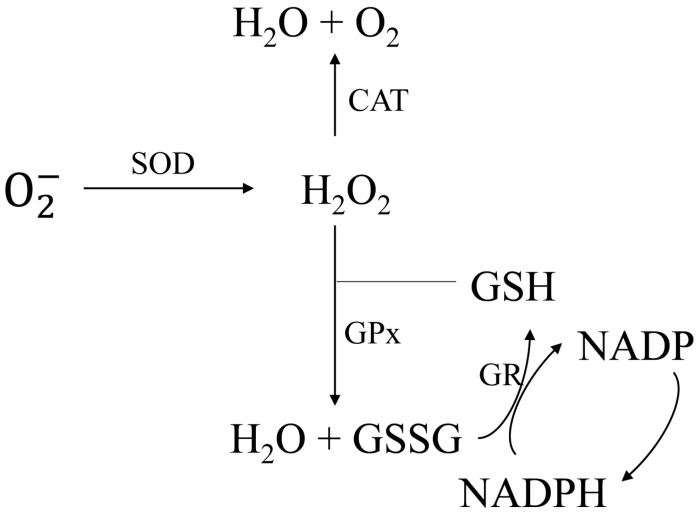
The antioxidant system in filamentous fungi. catalase, CAT; superoxide dismutase, SOD; glutathione peroxidase, GPx; glutathione reductase, GR; reduced glutathione, GSH.

Oxidative stress responses of several filamentous fungi have already been introduced, such as those of *Aspergillus niger* [12], *Neurospora crassa* [13], and *Phycomyces blakesleeanus* [14]. Excellent enzyme and non-enzyme systems (Figure 1) in fungi act in essential roles of eliminating oxidative stresses [8]. These systems ensure cells maintain redox homeostasis and normal physiological activity [10,15]. Antioxidant enzymes are thought to be essential responses to these oxidative stresses, and these proteins include catalase (CAT), superoxide dismutase (SOD), glutathione peroxidase (GPx), and glutathione reductase (GR). SOD can catalyze toxic superoxide anions to relatively less harmful H_2_O_2_. The conversion of H_2_O_2_ to H_2_O can be further carried out by CAT at high substrate-turnover rates [8]. The non-enzyme defense systems mainly contain antioxidants, such as reduced glutathione (GSH). With the synergistic action of GPx, GSH also has the ability to transform H_2_O_2_ to H_2_O. Coupling with reduced nicotinamide adenine dinucleotide phosphate (NADPH), the GR can maintain cellular GSH contents through the glutathione pathway [16].

Oxidative stress is also involved in secondary metabolite biosynthesis. Miranda et al. have reported the relationship between lovastatin biosynthesis and ROS content in submerged fermentation [17]. The β-carotene biosynthesis of *Blakeslea trispora* [18] is induced by oxidative stress from exogenous H_2_O_2_. As described by Zhang, et al. [19], hypocrellin biosynthesis is also stimulated by exogenous 30 μM H_2_O_2_, accompanied by an increase of CAT and SOD activities. However, the response of hypocrellin biosynthesis and biomasses of *S. bambusicola* to high concentration H_2_O_2_ have not yet been inferred.

This paper aims to study the biochemical responses of *Shiraia* sp. SUPER-H168 to oxidative stress mainly resulting from exogenously high concentrations of H_2_O_2_. Under these stresses, biochemical parameters including biomass and hypocrellin production were evaluated. We further tested variations of antioxidant enzyme activities and antioxidant content. Hypocrellin is known as a source of oxidative stress, so we also assessed the responses of a hypocrellin-deficient strain to exogenous oxidative stress.

## 2. Results

### 2.1. Effect of H_2_O_2_ on Hypocrellin Production and Growth

**Figure 2:**
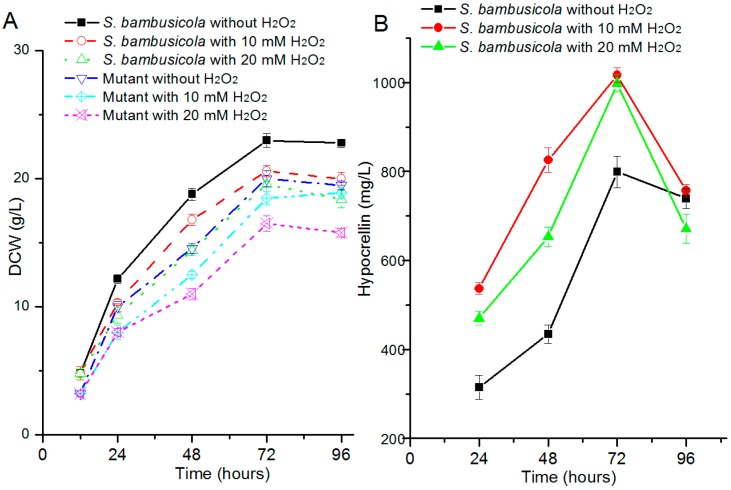
(**A**) Effects of H_2_O_2_ addition (0, 10, and 20 mM) on dry cell weight (DCW) of *S. bambusicola* and a hypocrellin-deficient mutant; (**B**) Effects of different concentrates of H_2_O_2_ on hypocrellin biosynthesis in *S. bambusicola*.

The effects of H_2_O_2_ addition on biomass and hypocrellin production were evaluated in *S. bambusicola*. The dry cell weight (DCW) (Figure 2A) of *S. bambusicola* slightly decreased when 10 mM H_2_O_2_ was added, compared with the control strain. On the other hand, when the concentration was up to 20 mM, the wild strain still kept a relatively high biomass and the value of the inhibition ratio was about 20%, which suggests that wild *Shiraia* sp. SUPER-H168 has a good tolerance for oxidative stress. The growth of the hypocrellin deficiency strain was slightly limited and presented similar trends to those of *S. bambusicola* in the oxidative stress assays (Figure 2A). The inhibition ratio of the hypocrellin deficiency strain was only 33% after 20 mM H_2_O_2_ addition.

The hypocrellin productions of *S. bambusicola* (Figure 2B) were remarkably increased in H_2_O_2_ inducing assays from 24 to 72 h (*p* < 0.001) and reached the maximum levels at 72 h. At that time, the hypocrellin yields were increased by 27% (10 mM H_2_O_2_) and 25% (20 mM H_2_O_2_) compared with wild *S. bambusicola*. Then the amounts of hypocrellin were obviously reduced in all assays of *S. bambusicola* after 72 h incubation.

### 2.2. Antioxidant Enzyme Activity Analysis

To analyze the roles of antioxidant enzymes in resisting environmental oxidative stresses, we monitored several antioxidant enzymes activities after high concentration hydrogen peroxide was added to the culture. These enzymes included SOD, CAT, GPx, and GR.

#### 2.2.1. Specific Activities of SOD and CAT

**Figure 3:**
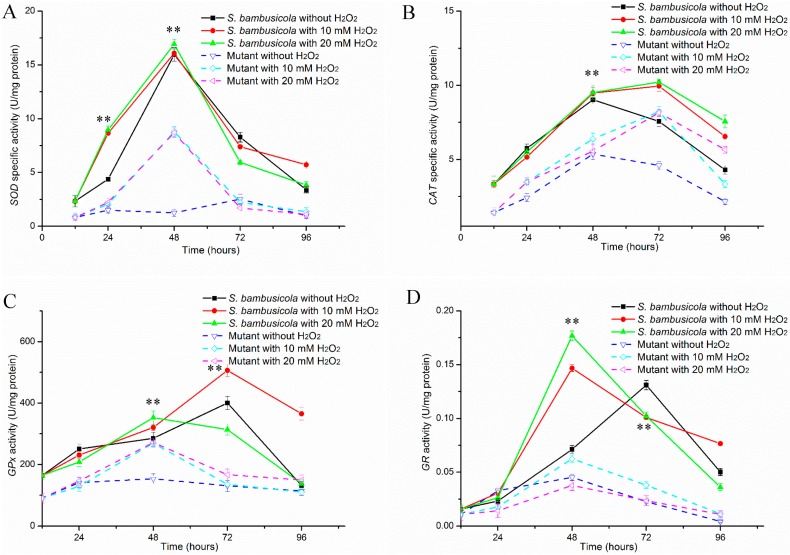
Effects of H_2_O_2_ addition on SOD (**A**); CAT (**B**); GPx (**C**); and GR (**D**) production of *S. bambusicola* and a hypocrellin-deficient mutant. ** *p* < 0.01.

Among all the samples except for the mutant without the H_2_O_2_ treatment, the SOD activities (Figure 3A) were improved at 12 h and reached the highest level at 48 h, then quickly reduced. The SOD activities of *S. bambusicola* with H_2_O_2_ treatment were significantly enhanced compared with the control at 24 h (*p* < 0.01). These SOD activities were improved by about one-fold at 24 h, and the quantities reached the maximum levels in all the samples of *S. bambusicola* at 48 h. Under H_2_O_2_ induction, the SOD production of the mutant was slightly increased at 24 h, then the values were remarkably improved at 48 h (*p* < 0.01) and increased by about seven times compared with the mutant without H_2_O_2_ treatment.

As it is a significant enzyme for scavenging oxidative stress, the CAT activities (Figure 3B) in the wild *S. bambusicola* and mutant were also assessed. The CAT activities of *S. bambusicola* with H_2_O_2_ were slightly inhibited at 24 h, then the values were rapidly improved at 48 h (*p* < 0.01) and reached the maximal amounts at 72 h. The activities of *S. bambusicola* with H_2_O_2_ were increased by about 30% compared with the control at 72 h, and the amounts still remained at higher levels at 96 h. Under higher concentrations of H_2_O_2_, the CAT yields of the mutant were significantly enhanced compared with the mutant without treatment at 24 h (*p* < 0.01). The CAT production of the mutant with H_2_O_2_ still kept an increasing trend at 48 h and reached the optimal levels at 72 h. Moreover, the CAT production with H_2_O_2_ treatment increased by about 80% when compared with the control.

In total, the CAT activities of a mutant treated with H_2_O_2_ represented higher levels than those of the none-treated mutant (*p* < 0.01). In addition, the *S. bambusicola* produced higher SOD and CAT yields than the mutant after H_2_O_2_ addition. Under H_2_O_2_ treatment, all maximal CAT yields from *S. bambusicola* and the mutant were obtained at 72 h, a delay of 24 h compared with the strains without H_2_O_2_ addition.

#### 2.2.2. Determinations of GPx and GR Activities

Figure 3C shows that *S. bambusicola* produced sufficient GPx, especially when higher hypocrellin production was obtained at 72 h. When H_2_O_2_ was supplied in culture, GPx activity in *S. bambusicola* was slightly less than the control at 24 h. Then GPx productions of *S. bambusicola* with H_2_O_2_ treatment rapidly improved at 48 h (*p* < 0.01). Under lower H_2_O_2_ (10 mM) inducing, the GPx yield of *S. bambusicola* successively improved to reach the optimal production at 72 h and increased by about 26% compared with the control (*p* < 0.01). It is worth noting that the GPx activities of *S. bambusicola* with 20 mM H_2_O_2_ treatment was downregulated at 72 h significantly more than the control. Unlike *S. bambusicola*, the GPx activity of the mutant kept at a steady level when no H_2_O_2_ was added to the culture and the time for optimal GPx was advanced to 48 h. In addition, the GPx production of the hypocrellin-deficient mutant with H_2_O_2_ was significantly upregulated at 48 h. The activities were increased by about 1.8 times compared with that of the control.

As another essential enzyme against oxidative stresses, GR activity was also measured. Figure 3D depicts that GR production of *S. bambusicola* was slightly enhanced by adding a high 20 mM H_2_O_2_ concentration. In addition, these GR outputs were more enhanced than the control *S. bambusicola* at 48 h (*p* < 0.01). The time of maximal GR productions advanced to 48 h sooner than with the control *S. bambusicola*. The GR yields of *S. bambusicola* were increased by one time (10 mM H_2_O_2_ treatment) and about 1.5 times (20 mM H_2_O_2_ treatment) at 48 h. Then these GR productions were quickly downregulated (*p* < 0.01) at 72 h. In addition, the GR activity of *S. bambusicola* treated with 10 mM H_2_O_2_ was still significantly enhanced compared with the control strain at 96 h (*p* = 0.002). Among the whole progress, the GR activities in the mutant with 20 mM H_2_O_2_ addition were more obviously downregulated than without H_2_O_2_ addition (*p* < 0.01). The GR yield in the mutant treated with 10 mM H_2_O_2_ underwent a little inhibition at 24 h, then the production was quickly improved at 48 h. The values remained at higher levels than without H_2_O_2_ addition, ranging from 48 to 96 h (*p* < 0.01).

### 2.3. GSH Content

**Figure 4:**
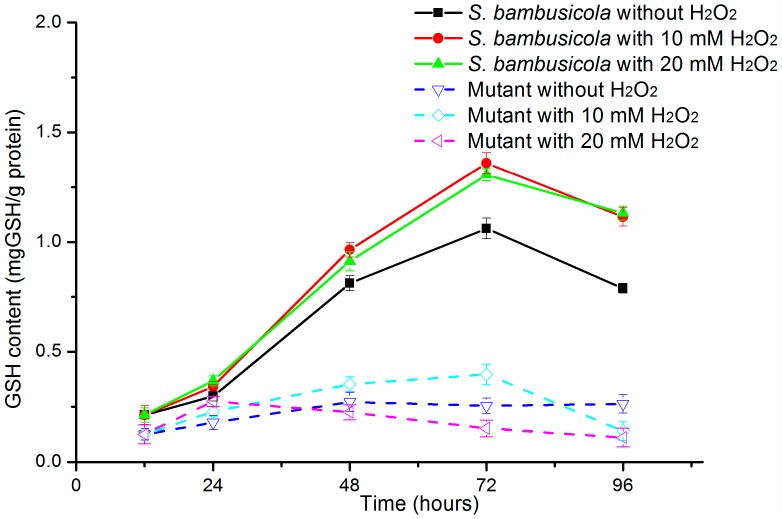
Effects of H_2_O_2_ addition on GSH contents of the *S. bambusicola* and the mutant.

As an essential antioxidant, the GSH content was also studied (Figure 4). Under different H_2_O_2_ concentrations, the GSH content of *S. bambusicola* showed a similar pattern of changes to the other antioxidant enzymes. In other words, the GSH productions of *S. bambusicola* with H_2_O_2_ treatment were significantly upregulated than in the control from 24 h to 96 h (*p* < 0.01). The values were improved by about 30% (10 mM H_2_O_2_) and 23% (20 mM H_2_O_2_) at 72 h. In all, with different concentrations of H_2_O_2_, the GSH yields of *S. bambusicola* were more abundant than in the mutant. Under H_2_O_2_ treatment, the GSH productions of the mutant were slightly enhanced compared to the mutant without treatment at 24 h. The GSH production of the mutant with 10 mM H_2_O_2_ treatment was successively improved compared with the mutant with no H_2_O_2_ treatment from 24 to 72 h (*p* < 0.01). However, under 20 mM H_2_O_2_ treatment, the GSH yields of the mutant were significantly limited from 24 to 96 h (*p* < 0.01).

## 3. Discussion

Among all ROS, H_2_O_2_ is less lethal for cells, and the stresses from exogenous H_2_O_2_ can stimulate metabolism biosynthesis of filamentous fungi. Under stress induced by H_2_O_2_, fumonisin yields of two *Fusarium verticillioides* [20] were increased more than two times by enhancing transcription levels of biosynthesis gene clusters. *Blakeslea trispora* also increased β-carotene levels by 38% under oxidative stress from 40 μM H_2_O_2_ [18]. Hypocrellin production was also increased from 110.0 mg/L to 408.5 mg/L after 30 μM H_2_O_2_ addition [19]. These increased products were mainly induced by lower concentration H_2_O_2_, while a few are increased with abundant H_2_O_2_ to stimulate metabolic biosynthesis. We found that high concentration H_2_O_2_ also had the ability to promote hypocrellin biosynthesis. The production (Figure 2) increased by ~27% when the concentration was 10 mM and improved by ~25% even under higher oxidative stress (20 mM). The DCW of *S. bambusicola* with 20 mM H_2_O_2_ treatment was only reduced by 20% compared with the control at 72 h. We could not help but ask how *S. bambusicola* could resist this oxidative stress. Hypocrellin is mainly obtained from stromata extraction of the parasitic fungi, such as *Hypocrella bambusae* and *S. bambusicola* [21,22]; however, yields of natural extraction were too low to meet its current medical demands. The enhancement of hypocrellin yield induced by additional H_2_O_2_ will relieve its shortage in medical applications.

The relationship between the regulation of oxidative stress and hypocrellin biosynthesis is still a puzzle. Montibus et al. [23] reviewed the relationship between oxidative stress response and secondary metabolism. Among the oxidative stress responses, transcriptional regulators are activated and are involved in the control of antioxidant machinery. *AtfB* [24], an oxidative stress-related bZIP transcription factor, is also involved in expression of antioxidant genes and aflatoxin biosynthesis. In a later study, we will further analyze the genome of *S. bambusicola* to find the transcription factors that are required for oxidative stress protection and hypocrellin metabolism. Then we will verify the function of the transcription factor by gene targeting.

Under illumination, hypocrellin can produce ROS including H_2_O_2_ and superoxide via type ‘‘type I’’ reaction and ^1^O_2_ via ‘‘type II’’ reaction. Abundant ROS will cause cellular oxidative stress [4]. This stress will usually damage cellular macromolecules. However, the DCW of *S. bambusicola* had a tendency to keep rising (Figure 2A) accompanied with an increase in hypocrellin [11]. Even when maximum hypocrellin production was obtained, the biomass remained at a constant level (Figure 2B). At this time, low ROS of *S. bambusicola* was measured in our previous work and the cell still retained redox homeostasis. It is suggested that *S. bambusicola* has excellent antioxidant systems to resist the environmental stresses. To maintain cellular ROS at a steady state, fungi have extraordinarily enzymatic and non-enzymatic systems to defend these detrimental oxidative stresses. These antioxidant enzyme systems also have been intensively discussed in filamentous fungi [12,14]. Similar to *A. niger* B1-D [12], the SOD activities of *S. bambusicola* and the mutant were rapidly enhanced for H_2_O_2_ detoxification after a burst of oxidative stresses (Figure 3A). In addition, there was a different pattern of change in CAT between *S. bambusicola* and the mutant. In detail, H_2_O_2_ addition quickly increased the CAT production of the mutant at 24 h, while it slightly inhibited the CAT yield of *S. bambusicola*. According to our knowledge, fungi generate three forms of CAT. These proteins are H_2_O_2_ inducible or constructive [25]. The different types of CAT guarantee *S. bambusicola* and its mutant have the ability to detoxify different environmental concentrations of H_2_O_2_ [14,25]. At 72 h, *S. bambusicola* still kept higher CAT yields against potential oxidative stress produced from maximum hypocrellin, which assured that *S. bambusicola* transformed toxic H_2_O_2_ to H_2_O. This could be one reason why *S. bambusicola* kept normal life activities when higher hypocrellin productions were obtained.

Another two essential antioxidant proteins, GPx and GR, of *S. bambusicola* play essential roles in defense from oxidative stress through the glutathione pathway [15,26]. The GR production is higher than GPx yield in *A. niger* B1-D [12], which ensure cells produce sufficient GSH to catalyze H_2_O_2_ to nontoxic H_2_O. However, in *S. bambusicola* and its mutant, greater GPx production was obtained than GR yields during the whole process. This discrepancy was also observed in *P. blakesleeanus* [14]. The redundant GPx ensure strains transform toxic H_2_O_2_ to nontoxic H_2_O, accompanied with successively producing GSH by the glutathione pathway [16], which suggests that GPx of *S. bambusicola* acts in an essential role in defense against oxidative stress. Therefore, *S. bambusicola* and its mutant have sufficient ability to avoid oxidative stress from exogenous 20 mM H_2_O_2_. At the same time, the enhanced GPx yields also guarantees *S. bambusicola* defends itself from the oxidative stresses of upregulated hypocrellin. The GR yields in *S. bambusicola* with H_2_O_2_ treatment were decreased at 72h than those of 48h, while higher GR productions were still detected at 72 h, which guaranteed abundant GSH content for H_2_O production from H_2_O_2_. It is well known that GSH is a significant non-enzymatic antioxidant in filamentous fungi and can reduce cellular oxidants, accompanying other non-enzymatic and enzymatic substrates [27,28]. In both *S. bambusicola* and its mutant, the GSH content was rapidly elevated under high amounts of H_2_O_2_ (Figure 3). Even under exogenous 20 mM H_2_O_2_, GSH yields still remained above 0.152 (mg GSH)/(g protein) in the mutant, similar to *P. blakesleeanus* [14], which ensured the conversion ability of abundant H_2_O_2_ to H_2_O. Overall, the antioxidant enzyme yields and GSH contents (Figure 4) of *S. bambusicola* were much higher than those of the mutant among different H_2_O_2_ treatment assays. In other words, wild *S. bambusicola* prominently represented oxidative stress tolerance. These results also explained that *S. bambusicola* had an excellent ability to resist higher concentration of H_2_O_2_ compared with the mutant. The excellent antioxidant systems in wild *S. bambusicola* also contribute to this strains tolerance against oxidative stresses from light-activated hypocrellin.

It is worth noting that aerobic fungi can generate H_2_O_2_ from incomplete reduction of oxygen. In oxygen-enriched fermentation processes, sufficient H_2_O_2_ is obtained to cause cellular oxidative stress [29], which disturbs the bioprocess. Based on its excellent oxidative stress tolerance, *S. bambusicola* can be potentially used as an industrial strain that is resistant to higher concentrations of H_2_O_2_ from oxygen-enriched fermentation. Therefore, this study can open up an avenue for industrial fermentation with higher endogenous or exogenous ROS.

In summary, *S. bambusicola* can retain normal physiology and hypocrellin biosynthesis is significantly stimulated after high concentration H_2_O_2_ treatment. The antioxidant enzymes, especially GPx and GR, and antioxidants act in essential roles in oxidative stress protection. These excellent antioxidant responses from *S. bambusicola* and its hypocrellin mutant to oxidative stresses ensure this fungus maintains physiological activities.

## 4. Experimental Section

### 4.1. Strain and Cultivation

*Shiraia* sp. SUPER-H168 (CCTCC M 207104) [3] and its hypocrellin-deficient strain were cultured in complete medium (CM medium) at 30 °C and 200 rpm. The composition of CM medium was as follows: 20 g/L glucose, 1 g/L K_2_HPO_4_, 0.5 g/L MgSO_4_·7H_2_O, 0.5 g/L KCl, 0.01 g/L FeSO_4_·7H_2_O, 3 g/L yeast extract, 10 g/L peptone, 200 g/L potato extract, and 0.6% Triton X-100. Different concentrations of H_2_O_2_ (0, 10, 20 mM) were added to batch cultures after 12 h incubation (the logarithmic phase). No H_2_O_2_ was added into the wild *Shiraia* sp. SUPER-H168 culture was chosen as the control.

In addition, the hypocrellin is known as a source of oxidative stress [4], therefore, the response of a hypocrellin-deficient strains to high concentrations H_2_O_2_ was also assessed. In addition, Newman and Townsend [30] have recently confirmed the biosynthesis pathway of cercosporin, which is a similar perylenequinone to hypocrellin. Moreover, among the genes for hypocrellin biosynthesis, polyketide synthase (*PKS*) has an essential role in biosynthesizing the carbon backbones of hypocrellin via repetitive decarboxylative claisen condensation [31,32]. In our previous work, a polyketide synthase deficient strain that cannot produce hypocrellin was obtained by clustered regularly interspaced short palindromic repeat sequences (CRISPR)/Cas9 method.

### 4.2. Biochemical Assays

The biomasses and hypocrellin productions were detected as described by Cai, Liao, Liang, Ding, Sun and Zhang [11]. A relative inhibition was used to evaluate the effects of H_2_O_2_ addition on the strain. It was calculated as the ratio of the (dry cell weight of sample strain)/(dry cell weight of control strain). The wild *S. bambusicola* without H_2_O_2_ treatment was used as a control.

The GSH contents [33] and the activities of the enzymes, including CAT [34], SOD [35], GPx [36], and GR [37] were tested as described previously. The results were measured from three independent experiments and expressed as mean ± SD.

## 5. Statistical Analysis

All statistical analyses were performed by SPSS 11.0 (IBM, New York, NY, USA). The statistical significance of the difference between the control sample and strains with H_2_O_2_ treatment was evaluated by paired sample t test. Statistical significance was established at *p* < 0.05.

## 6. Conclusions

Hypocrellin production of *S. bambusicola* can be significantly improved after higher concentration H_2_O_2_ treatments (10 and 20 mM). *S. bambusicola* can retain normal physiology under these oxidative stress. The antioxidant enzymes, especially GPx and GR, and antioxidants act in essential roles in the oxidative stress protection. These excellent antioxidant responses from *S. bambusicola* and its hypocrellin mutant to oxidative stresses ensure this fungus maintains physiological activities.
